# Successful treatment of pyrotinib for bone marrow metastasis induced pancytopenia in a patient with non‐small‐cell lung cancer and *ERBB2* mutation

**DOI:** 10.1111/1759-7714.13480

**Published:** 2020-05-26

**Authors:** Yanyan Wu, Jun Ni, Xiaoyan Chang, Xiaotong Zhang, Li Zhang

**Affiliations:** ^1^ Department of Pulmonary and Critical Care Medicine, Peking Union Medical College Hospital Chinese Academy of Medical Sciences & Peking Union Medical College Beijing China; ^2^ Department of Pathology, Peking Union Medical College Hospital Chinese Academy of Medical Sciences & Peking Union Medical College Beijing China

**Keywords:** Bone marrow metastasis, *ERBB2* mutation, NSCLC, pyrotinib

## Abstract

*ERBB2* mutations are found in about 2% of patients with non‐small cell lung cancer (NSCLC). A recent study reported that pyrotinib (an irreversible pan ErbB inhibitor) had superior antitumor effect compared to other tyrosine kinase inhibitor therapies in patients with *ERBB2* mutations. Bone marrow metastasis is rare in lung adenocarcinoma, and has been reported to be associated with poor prognosis. Here, we report the case of a 62‐year‐old female diagnosed with lung adenocarcinoma and bone marrow metastasis. *ERBB2* exon 20 insertion mutation was confirmed by next‐generation sequencing (NGS) of lung tissue as well as bone marrow. The patient achieved stable disease and recovery of pancytopenia after two months of pyrotinib therapy. This is the first report of homogenous mutations of *ERBB2* detected in bone marrow, as well as a good response of bone marrow to pyrotinib therapy.

**Key points:**

- This is the first report of a homogenous mutation of *ERBB2* detected in the bone marrow of an NSCLC patient with bone marrow metastasis.
- Our patient with NSCLC *ERBB2* mutation and bone marrow metastasis responded well to pyrotinib therapy.

## Introduction

Human epidermal growth factor receptor 2 mutations (*HER2*, *ERBB2*) have been found in about 2% of patients with non‐small cell lung cancer (NSCLC) 1, 2. However, in the past 10 years, effective targeted therapies have not been identified. Recently, Wang *et al*.^3^ reported that pyrotinib, an irreversible pan ErbB inhibitor, provided an overall response rate of 53.5% in a phase 2 study. Bone marrow metastasis is rare in patients with lung adenocarcinoma, and has been reported to be associated with poor prognosis. Here, we present a rare NSCLC case with bone marrow metastasis carrying *ERBB2* mutations which responded well to pyrotinib therapy.

## Case presentation

**Figure 1:**
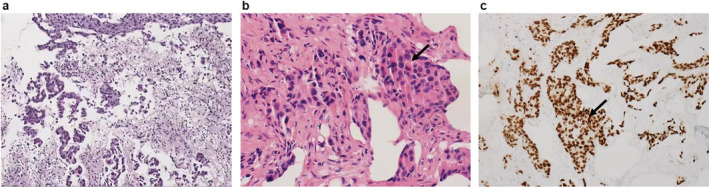
Lung and bone marrow biopsy pathology of the patient. (**a**) Lung biopsy pathology at diagnosis, May 2018; (**b**) bone marrow biopsy pathology in August 2019, metastatic adenocarcinoma was seen in the bone marrow of the patient. Metastatic adenocarcinoma cells are indicated with an arrow; and (**c**) immunohistochemical result showed that metastatic adenocarcinoma cells in bone marrow were positive for TTF‐1.

**Figure 2:**
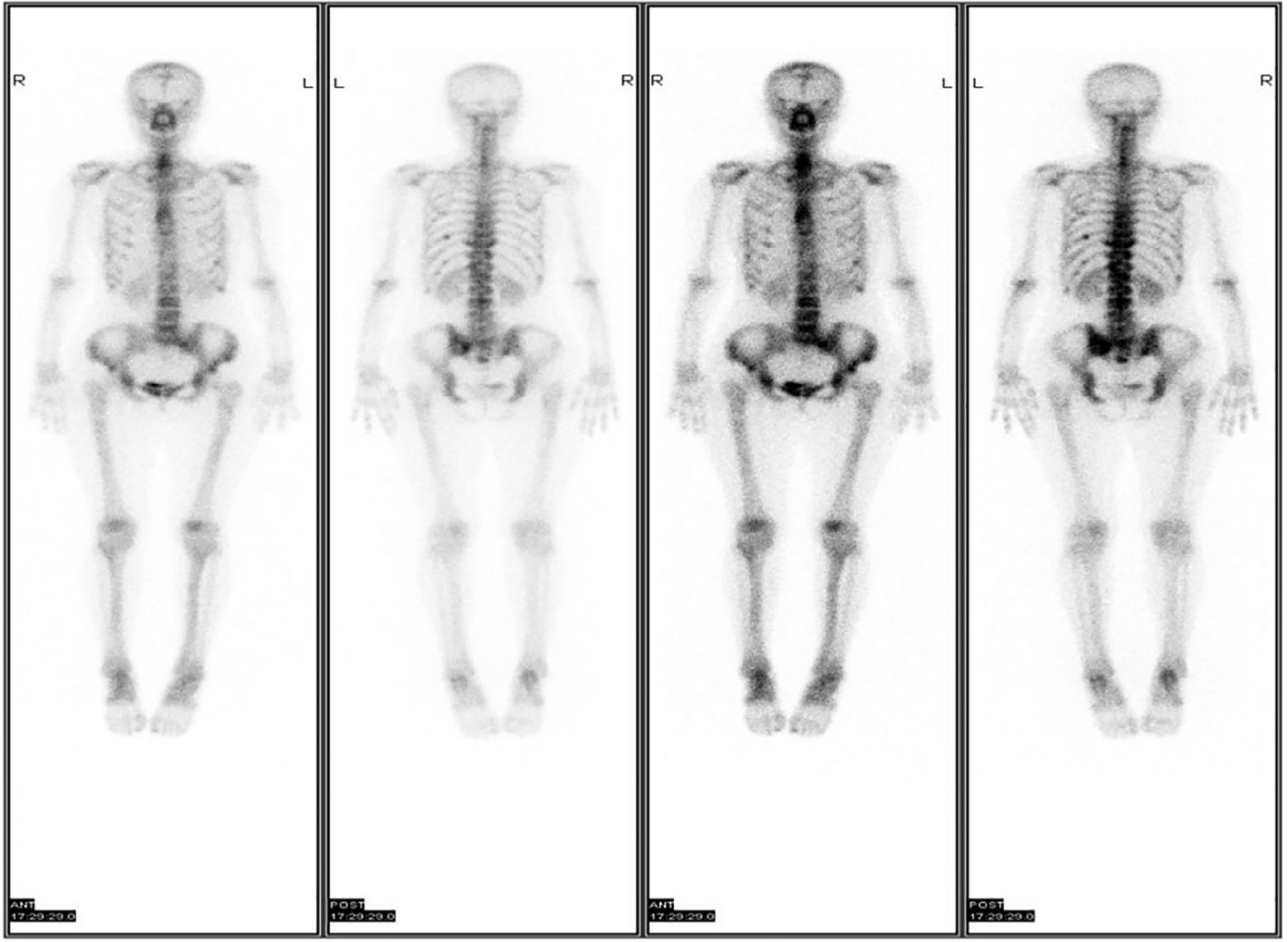
Bone scan of the patient. Increased uptake was detected in multiple ribs, spines, pelvic bones and bilateral sacroiliac joints.

**Figure 3:**
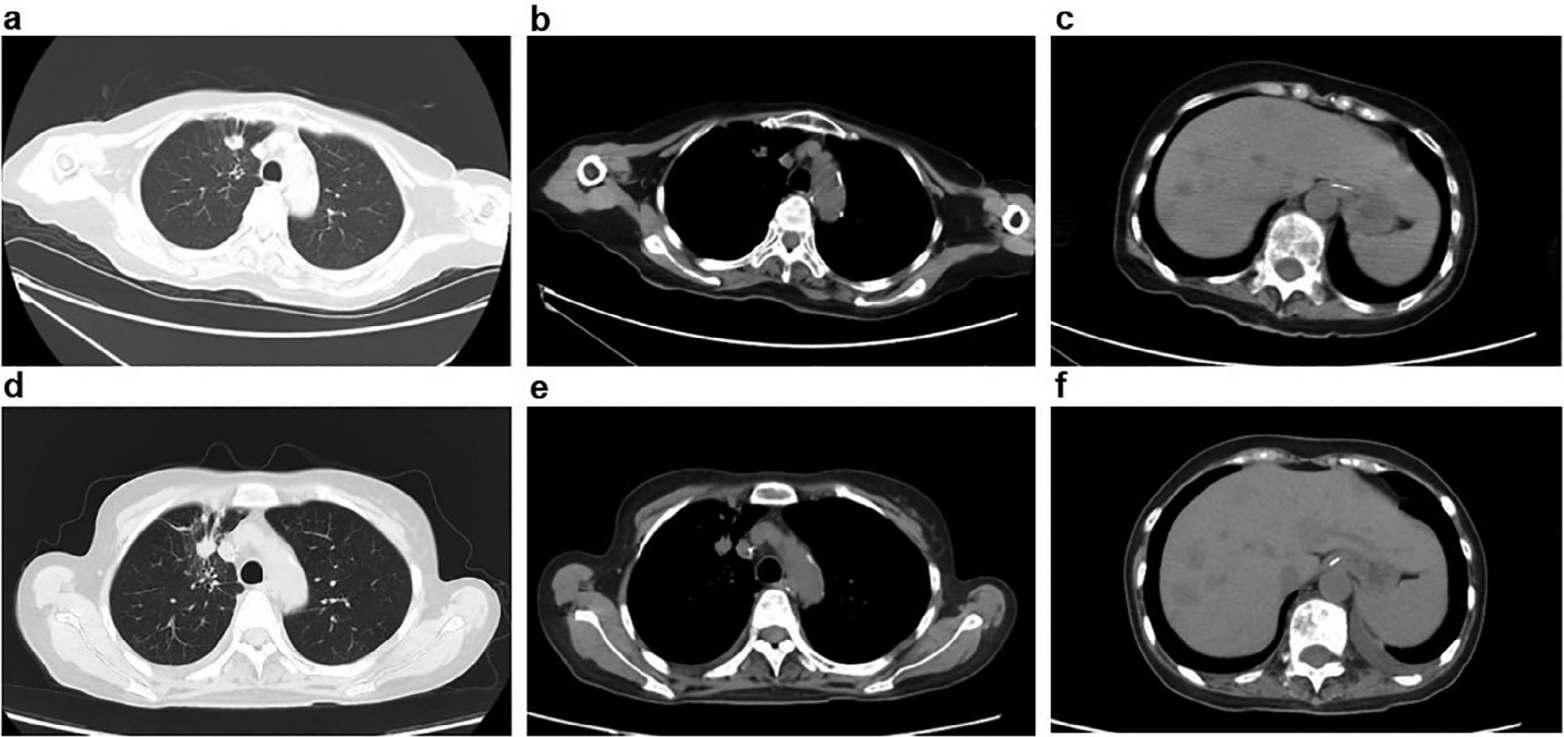
Chest and abdominal radiograph of the patient. (**a**, **b**, **c**) Chest abdominal computed tomography (CT) scan on 6 August 2019, the size of the target lesion 1.2 cm × 1.0 cm, liver metastasis, after one cycle of anlotinib; (**d**, **e**, **f**) chest and abdominal CT scan on 16 October 2019, the size of the target lesion 1.3 cm × 1.1 cm, liver metastasis, after two months of treatment of pyrotinib.

A 62‐year‐old woman (non‐smoker) was diagnosed with stage IIIb NSCLC in May 2018. Lung histopathology confirmed adenocarcinoma (Fig 1a). Next‐generation sequencing (NGS) showed an *ERBB2* exon 20 insertion mutation (p.E770delinsEAYVM 24.34%) and a *TP53* mutation (p.S241F 10.98%), with a PD‐L1 tumor proportion score less than 1%. The patient received six cycles of chemotherapy (pemetrexed/cisplatin) combined with pembrolizumab, underwent radiotherapy of the right lung lesion (64 Gy/eight fractions, three fractions a week) and received six cycles of pemetrexed afterwards as maintenance therapy. Clinical response was evaluated every two cycles as stable disease. However, after six cycles of maintenance therapy, multiple bone metastatic lesions were detected on bone scan in June 2019 (Fig 2). The patient refused further chemotherapy and received one cycle of anlotinib (a multitargeting tyrosine kinase inhibitor and angiogenesis inhibitor) without any improvement. On 6 August 2019, she was unconscious and was admitted to hospital. Blood tests showed anemia, thrombocytopenia, hypercalcemia, and elevation of creatinine. Coagulation profile suggested disseminated intravascular coagulation (DIC). Several serum tumor markers were significantly elevated (Table 1). Hepatic metastases was confirmed by computed tomography (CT) scan (Fig 3a–c). Bone marrow biopsy suggested bone marrow metastasis (Fig 1b,c). The NGS of bone marrow biopsy revealed the copy number alteration of *ERBB2* gene (*n* = 3.34) and an *ERBB2* exon 20 insertion mutation as previously described (p.Y772_A775dupYVMA 38.45%).

**Table 1:** Laboratory data of the patient

Blood tests	Reference range, adult female	6 August On admission	18 August Before pyrotinib therapy	23 August Five days after pyrotinib therapy	17 September One month after pyrotinib therapy	16 October Two months after pyrotinib therapy
Complete blood count						
WBC count, ×10^9^/L	3.5–9.5	11.4	8.3	5.6	4.4	4.3
NEUT count, ×10^9^/L	2.0–7.5	7.9	7.4	3.7	2.7	2.8
HGB, g/L	110–150	100	71	74	72	92
PLT count, ×10^9^/L	100–300	31	22	48	220	268
Electrolytes						
Sodium, mmol/L	135–145	160	136	136	137	138
Potassium, mmol/L	3.5–5.5	3.7	3.6	4.2	4.0	5.0
Chloride, mmol/L	96–111	113	97	102	102	103
Calcium, mmol/L	2.13–2.70	4.61	3.19	2.09	2.09	2.19
Creatinine, umol/L	45–84	267	141	80	62	56
Coagulation function						
PT, s	10.4–12.6	13.9	13.3	12.3	12.5	–
APTT, s	23.3–32.5	34.7	29.8	29.4	25.4	–
Fbg, g/L	1.8–3.5	1.4	1.9	2.8	4.0	–
D‐Dimer, mg/L FEU	0–0.55	19.62	40.41	24.67	4.25	–
FDP, umol/L	0–5.0	28.96	217.3	76.6	10.7	–
Serum tumor markers						
CA19‐9, U/mL	0–34.0	57.3	–	103.9	62.9	65.3
CEA, U/mL	0–5.0	5851.0	–	4455.0	5413.0	3823.0
CA125, U/mL	0–35.0	629.0	–	594.3	261.2	408.6
CYFRA 21‐1, U/mL	0–3.5	–	–	14.9	4.9	8.3
CA242, U/mL	0–20.0	65.6	–	56.0	47.8	47.7
NSE, U/mL	0–16.3	99.4	–	24.7	13.9	25.0
CA72‐4, U/mL	0–9.8	290.2	–	353.2	12.9	14.6
CA15‐3, U/mL	0–25.0	35.4	–	45.6	29.3	24.6
SCCAg, U/mL	0–2.7	1.6	–	1.1	0.8	1.0
proGRP, U/mL	0–69.2	259	–	211	250	148

APTT, activated partial thromboplastin time; CA, carbohydrate antigen; CEA, carcinoembryonic antigen; CYFRA21‐1, cytokeratin 19 fragments; Fbg, fibrinogen; FDP, fibrinogen degradation product; HGB, hemoglobin; NEUT, neutrophil; NSE, neuron‐specific enolase; PLT, platelet; proGRP, gastrin‐releasing peptide precursor; PT, prothrombin time; SCCAg, squamous cell carcinoma antigen; WBC, white blood cells.

On 18 August 2019, pyrotinib therapy (240 mg q.d.) was initiated, based on *ERBB2* exon 20 insertion mutation. One month later, platelet count, renal function and coagulation function of the patient had returned to normal and pyrotinib dose was subsequently increased to 320 mg q.d. Two months later on 16 October 2019, she was assessed as stable disease (Table 1, Fig 3d–f).

## Discussion

With the discovery of driver mutations such as epidermal growth factor receptor (*EGFR*) and the development of tyrosine kinase inhibitor (TKI) therapies targeting these mutations, the treatment of NSCLC has moved from conventional chemotherapy to targeted therapies. Human epidermal growth factor receptor 2 mutations (HER2, *ERBB2*) are found in about 2% of NSCLC, with a predominance in women and non‐smokers^1^, ^4^, ^5^. About 96% of *ERBB2* mutations are exon 20 insertions, and 83% of those are a recurrent 12 base‐pair insertion causing duplication of amino acids YVMA at codon 775.^2^, ^6^ Patients with *ERBB2* mutations have previously shown a low response to pemetrexed‐based first‐line chemotherapy.^7^ Immunotherapy achieved limited efficacy in these patients with response rates varying from 7% to 27%.^8^, ^9^ Since 2006, several case reports and case series have suggested transtuzumab, afatinib, dacomitinib and neratinib as potential targeted therapies for patients with NSCLC carrying *ERBB2* mutations.^10^, ^11^, ^12^, ^13^ Early clinical activity has also been seen with the dual EGFR^14^ TKIs, poziotinib and TAK‐788. Mazières *et al*. have recently reported 65 patients receiving *ERBB2*‐targeted therapies (trastuzumab and afatinib) with an overall response rate (ORR) of 50.9% and a median PFS of 4.8 months^15^. In 2018, Wang *et al*. reported pyrotinib, as a pan HER receptor tyrosine kinase inhibitor, showed superior antitumor effect than afatinib and trastuzumab in vitro. In a phase 2 study of 15 patients, treatment with pyrotinib provided an ORR of 53.5% and a median PFS of 6.4 months.^3^ In our case, the patient carried an exon 20 insertion leading to duplication of amino acid YVMA at codon 775 in *ERBB2*, which is the most often seen mutation in NSCLC.^2^ She was given pyrotinib 240 mg q.d. first for renal insufficiency, and the dose was adjusted to 320 mg q.d. The patient achieved stable disease and recovered from acute kidney injury and bone marrow depression, which is in accordance with the report by Wang *et al*.^3^

Bone marrow metastasis is rarely reported in NSCLC. Tumor cells invading bone marrow will lead to pancytopenia and hematological disorders such as DIC and microangiopathic hemolytic anemia. In our case, it is first reported that the NGS of bone marrow confirmed homeotic mutations with lung tissue and the patient recovered from severe anemia, thrombocytopenia and DIC after pyrotinib therapy.

## Disclosure

The authors declare there are no conflicts of interest.
